# The Lime-Juice Ration

**Published:** 1900-05-26

**Authors:** 


### The Lime-juice Ration.

Although still now and again met with in ordinary practice, scurvy has long since been deposed from the important position which it used to hold in the nosology. Time was when the influence of a maritime power might be seriously crippled by outbreaks of scurvy in her fleets, and when this disease exercised a definite control over the length of sea voyages. On land also scurvy has frequently been a factor of not inconsiderable importance in the making of history, by diminishing the efficiency of armies in the field and by destroying those shut up in besieged cities. But in the normal circumstances of civilised life scurvy is now practically a disease of the past, except where it breaks out as a complication of rickets in children who have been deprived of their natural food.

It has long been an article of medical faith that the cause of the scurvy which used to be so common among sailors was the absence of vegetable food from the dietary, and that its practical eradication has been due to the use of antiscorbutics, and especially of the daily dose of lime-juice. Indeed, if there is one thing that is held to be more certain than another it is that (to quote from Allbutt's "System of Medicine") "the only sure and certain effectual means of preventing this disease, and of curing it when it has shown itself, is the supply of fresh succulent vegetables or fruits, or of a pure vegetable juice.

Some years ago, however, certain conditions met with in infants were shown to be identical with true scurvy, and curable by the same means. As Dr. Cheadle justly says, "there is nothing in the whole range of medicine . . . . more striking and remarkable than the immediate and rapid recovery which follows the administration of fresh vegetable material and other fresh elements of food in these cases of infantile scurvy." Yet the fact that the disease attacked infants, often at an age before vegetables usually enter into a child's diet, and the further fact that the only departure from the normal in the feeding of some of these cases seemed to have consisted of an absence of fresh milk, tended to suggest the importance of the "freshness" rather than the vegetable nature of the food in the prevention and treatment of scorbutic conditions. Thus we began to talk of "living food," and to suspect that some useful element in the milk might be killed by the processes of "sterilisation" to which cow's milk is often subjected. The idea that it was freshness rather than vegetable character which was the essential thing, was strongly reinforced by the experiences of Nansen and Johansen, who, although entirely without vegetables, and even without lime-juice, and although they passed their time almost constantly in the dark, without exercise, and in the midst of most insanitary conditions, yet escaped scurvy, apparently in consequence of having lived exclusively on walrus meat or bear's meat, which froze directly it was killed, and had thus been exposed neither to decomposition nor to preserving processes. The same thing was shown by an observation made by Frederick G. Jackson when living among the Samoyards on Waigatz Island. These people never take vegetables and know not lime-juice, yet scurvy does not occur among them. They, however, live entirely upon fresh reindeer meat. On the other hand, those who in the autumn migrate south, with the Russian traders, and live, as is usual with them, upon salted fish, suffer much from that disease. What, then, are we to think of our old faiths? and especially what are we to think of the lime-juice which for now over a hundred years has been served out so punctiliously to our sailors to keep off scurvy? Is this a mere fetich? This is a question which has been taken up by Mr. Frederick G. Jackson and Dr. Vaughan Harley in a paper recently read before the Royal Society. They show, from the experience gained in the Nares Polar Expedition, in which the crews of both the "Alert" and the "Discovery" suffered greatly from scurvy, even though lime-juice was taken daily by all on board, that lime-juice does not always have the antiscorbutic effect so commonly attributed to it; and, on the other hand, they insist upon the fact mentioned above, that those who live on fresh meat escape scurvy, even although they do not take either vegetables or lime-juice, and they maintain that the real and essential cause of the disease is the consumption of *tainted food*. They added to the diet of one set of monkeys meat from a freshly-opened tin, and these did not get scurvy. To the diet of another set they added meat from a tin that had been opened some days, and had stood in the laboratory. It was not what one would call "bad," but it had a distinctly sour smell. These monkeys developed a disease which it seems impossible to doubt was scurvy. The third set of monkeys were treated in the same manner as the last, but in addition they received either an apple or a banana every day. They also got scurvy, although not in the same degree. We do not suggest that these investigations solve the problem, which is far too important a one to be allowed to hinge on any two or three dozen experiments; but, so far as they go, they strongly confirm the opinion which these authors hold, that scurvy is a form of chronic ptomaine poisoning produced by the eating of tainted food, and not a disease caused by the want of some hypothetical antiscorbutic material contained in fresh vegetables; and that, before giving to lime-juice the credit of having practically swept away scurvy from the naval and the marine services, it is necessary to remember that other causes have at the same time been at work to promote health, such as improved sanitation, better quarters, shorter voyages, and, above all, better food.

